# Reversible acute blindness in suspected metformin-associated lactic acidosis: a case report

**DOI:** 10.1186/s13256-023-04219-y

**Published:** 2023-11-23

**Authors:** Rui Huang, Wentao Sun

**Affiliations:** 1 Department of Emergency Medicine, The First Affiliated Hospital of Kunming Medical University, Kunming, 650000 People’s Republic of China; 2 Department of Plastic Surgery, The First Affiliated Hospital of Kunming Medical University, Kunming, 650000 People’s Republic of China

**Keywords:** Metformin, Blindness, Lactic acidosis

## Abstract

**Background:**

Metformin is commonly used for the treatment of type 2 diabetes mellitus. Its multiple advantages include low risk of hypoglycemia, weight neutrality, low cost, and cardioprotective and anti-inflammatory effects. Renal insufficiency is one of the contraindications for its use. Inadvertent prescription in patients with renal insufficiency may lead to metformin-associated lactic acidosis, which brings a high risk of mortality. The early recognition and management of metformin-associated lactic acidosis are essential.

**Case report:**

We present the case of a 58-year-old Hui woman with a history of type 2 diabetes mellitus with nephropathy and heart disease for which she was treated with metformin, insulin, and heart medications. She developed nausea, vomiting, anion gap metabolic acidosis due to hyperlactatemia, and acute kidney injury. She was hospitalized to receive intravenous hydration and correction of metabolic acidosis after she suddenly developed blindness. The diagnostic workup ruled out central causes and her symptoms resolved briefly after continuous venovenous hemodialysis was initiated, confirming the diagnosis of metformin-associated lactic acidosis.

**Conclusions:**

Metabolic disruption can cause acute blindness. Metabolic acidosis in a patient with a history of metformin intake should suggest the possibility of metformin-associated lactic acidosis, which must be treated immediately, without waiting for the results of other examinations, especially in patients with sudden blindness. Further study of reversible blindness-associated severe metabolic acidosis is needed.

## Introduction

Metformin is a biguanide used as a first-line medication to treat type 2 diabetes mellitus. Its use in patients with decreased renal function may lead to metformin-associated lactic acidosis (MALA) [1–3]. Historically, metformin was not recommended for use in patients with an estimated glomerular filtration rate (eGFR) < 60 mL/minute/1.73 m^2^, but in 2016 the US Food and Drug Administration (FDA) recommended the use of metformin even in patients with eGFR of 30–60 mL/minute/1.73 m^2^ [4].

States of severe metabolic acidosis are usually accompanied by unstable vital signs and high mortality [1–3, 5]. Therefore, determination of the cause of acidosis has an impact on patient prognosis. Severe metabolic acidosis may cause transient blindness in a subset of cases [6–9]. Diabetic acidosis, alcoholic ketoacidosis, and MALA are the most commonly encountered causes of reversible blindness associated with acidosis. The mechanism of metabolic acidosis-induced transient and reversible blindness has not yet been clearly identified [10–12].

In previous reports, unstable vital signs were usually noted along with the blindness of metabolic acidosis cases [6–9]. In a recent patient of ours, the initial vital signs were relatively stable, except for the rapid respiratory rate. In routine practice, this would usually indicate blindness from cerebrovascular or ocular disease and consequently delay the management of metabolic acidosis, with fatal consequences. We present the case of a 58-year-old woman with acute-onset blindness due to severe metabolic lactic acidosis.

## Case report

A 58-year-old Hui woman who complained of nausea and vomiting for 3 days and acute painless bilateral vision loss for 4 hours was admitted to the hospital on 26 May 2020 at 12:30. After admission, informed consent was obtained from the patient to confirm her approval for participation in the study.

Review of systems was negative for trauma, headache, cerebrovascular disease or vasculitides, eye pain, focal weakness or numbness, recent illness, overdose of any medication, or ingestion of xenobiotics other than her prescribed medications. She and her family denied any history of drug abuse, including methanol, salicylate, ethylene glycol, and isopropyl alcohol.

She was of the Hui ethnic group and retained the custom of eating only two meals (before sunrise and after sunset) each day during the traditional Ramadan month (from 24 April to 24 May 2020). She adjusted the frequency of subcutaneous injections of aspart insulin (from three times daily to twice daily) due to the change in her eating habits, but she did not monitor her blood glucose concentration, nor did she adjust her dose of metformin.

The patient’s medical history was significant for type 2 diabetes mellitus, hypertension, chronic heart failure, and chronic kidney disease. She had suffered from type 2 diabetes and hypertension for more than 10 years, cardiac enlargement for 6 years, and chronic kidney disease for several years. For her diabetes, her medications included metformin 1000 mg three times daily and aspart insulin 10 units subcutaneously three times daily before meals. For hypertension, her medications included amlodipine besylate 7.5 mg daily and irbesartan/hydrochlorothiazide 150/12.5 mg daily. For chronic heart failure, her medications included torasemide 20 mg twice daily, spironolactone 20 mg daily, and metoprolol succinate 47.5 mg daily. For her kidney disease, she did not take any special medication (blood creatinine concentration fluctuated between 1.58 and 1.70 mg/dL, eGFR 1.58–1.70 mL/minute/1.73 m^2^, chronic kidney disease (CKD) stage 3).

On presentation, her initial vital signs were a temperature of 36.6 °C, blood pressure of 129/80 mmHg, heart rate of 87 beats per minute, and respiratory rate of 29 breaths per minute. Oxygen saturation was normal on ambient air. The patient was alert and oriented and complained of only mild abdominal discomfort and blindness. She had no signs of trauma to the head or body. Her breathing was shallow and fast, the breathing sound in both lungs was thick, and wet rales could be heard in both lower lungs. No abnormal signs were found on heart or abdominal physical examination. Her limbs were wet and cold. No focal neurological deficits except ocular findings were noted. Her vision was impaired, and she could not perceive any light or hand movements. Both pupils were equal in size (3 mm), but the direct and indirect light reflexes were obviously dulled. Fundoscopic examination revealed bilateral diabetic retinopathy, which was not severe enough to cause acute blindness.

The patient’s laboratory data are listed in Table 1. Severe high-anion-gap metabolic lactic acidosis was noted, with associated renal insufficiency. Routine blood tests showed a high white blood cell count (23.65 × 10^9^/L) (neutrophils 0.74, red blood cells 4.07 × 10^12^/L, hemoglobin 122 g/L, platelets 294 × 10^9^/L). Cerebral computed tomography (CT) for evaluation of ocular, periocular, and intracranial lesions revealed nothing remarkable. The blood levels of ethanol, methanol, ethylene glycol, isopropyl alcohol, and metformin could not be estimated owing to the lack of these facilities at our hospital.

**Table 1:** Laboratory values during patient hospitalization

	0 hours on admission	2 hours	7 hours hemodialysis initiation	10 hours	13 hours vision recovery	24 hours	36 hours	16 days
Arterial blood gas								
pH	6.8	6.83	6.84	6.98	7.02	7.29	7.32	7.45
pO_2_ (mmHg)	145	109	64	154	52	50	61	80
pCO_2_ (mmHg)	14	14	15	15	23	20	55	34
Bicarbonate (mmol/L)		< 3	< 3.0	3.5	5.9	9.6	28.3	23.6
Base excess (mmol/L)		−29.1	−29.8	−26.2	−25.1	−15.2	1.6	−0.4
GAP anion (mmol/L)		52	56	48	42	23	20	7
Glucose (mg/dL)	90	104.4	135	144	158	165.6	169.2	158.4
Lactate (mmol/L)	> 20	> 20	> 20	> 20	17.4	2.7	1.2	0.9
Sodium (mEq/L)	142	138	145	144	143	141	153	135
Potassium (mEq/L)	6	5.7	3.6	4.1	4.6	3.3	3.2	4
Renal function parameters								
Creatinine (mg/dL)	11.22					4.0		3.09
Urea (mg/dL)	653.76					114.48		317.34

The initial management included treatment for acidosis with intravenous crystalloids and sodium bicarbonate, with control of blood glucose. Two hours after that treatment, the result of blood gas analysis showed that the acidosis was not significantly relieved, so she was transferred to the Emergency Intensive Care Unit (EICU). Seven hours after admission, continuous venovenous hemodialysis (CVVHD, 150 mL/hour) was performed via the right femoral vein using a Fresenius 4008S dialysis machine and Fresenius Ultraflux AV600S dialyzers (Fresenius Medical Care AG, Bad Homburg, Germany). The dialysate flow was 150 mL/minute. On the 13th hour after admission (6 hours after hemodialysis), although her blood pH value had not been completely corrected to normal (from 6.8 to 7.02), her visual acuity and light reflexes had significantly recovered (Table 1). On the 7th day after admission, the acidosis, lactic acid, and urine output of the patient were all normal, though blood creatinine fluctuated between 3.10 and 3.28 mg/dL. Therefore, hemodialysis was stopped, and she was admitted to the normal ward for conservative management and control of blood glucose.

She was discharged with her usual vision and overall condition 24 days after admission and has been under the management of the endocrinology and ophthalmology outpatient clinics at our institute.

## Discussion

Metformin, a biguanide oral antihyperglycemic, was approved for use in the USA in 1992 for the treatment of type 2 diabetes mellitus. Since its introduction, MALA has become a well-recognized adverse event [1–3], particularly when administered to patients with decreased renal function. Diabetic patients who are treated with renin–angiotensin system blockers and develop volume depletion and/or are given a nonsteroidal anti-inflammatory drug can quickly develop acute kidney injury, precipitating severe lactic acidosis in the setting of metformin therapy. Under those circumstances, it can accumulate to toxic levels and generate MALA [7]. MALA is extremely rare, with an estimated incidence of 0.03–0.06 per 1000 patient-years, based on spontaneous case reports [13]. Most cases occur in patients with predisposing conditions or intercurrent precipitating events that predispose them to lactic acidosis [13].

Lactic acidosis resulting from metformin toxicity should be suspected in any patient meeting all of the following criteria: a history of metformin administration; severe acidemia (pH 7.1); a markedly elevated lactate level (> 15 mmol/L) with a large anion gap (> 20 mmol/L); a very low serum bicarbonate level (< 10 mmol/L); and a history of renal insufficiency (glomerular filtration rate < 45 mL/minute or serum creatinine level > 2.0 mg/dL) [14]. The diagnostic criteria of MALA include the measurement of plasma concentrations of metformin, but few hospitals have adequate infrastructure, and this test is also time-consuming[15]. It is therefore impossible to measure blood concentrations of metformin in routine clinical practice. Consequently, MALA should be suspected in patients with a history of metformin intake, irrespective of whether their plasma levels of metformin are known [15–18]. In our case, although the plasma concentration of metformin was not estimated, treatment for MALA was instituted immediately on the basis of the history of metformin intake, the presence of metabolic acidosis, and renal insufficiency, with successful results.

MALA is more likely to occur in patients who acutely develop renal impairment from dehydration, vomiting, or diarrhea, especially in elderly subjects who have a low glomerular filtration rate [13]. According to one estimate, approximately 25% of patients taking metformin have one or more contraindications to it [19]. Our patient was of the Hui ethnic group and followed the custom of eating only two meals during the traditional Ramadan month. Failure to eat and frequent vomiting for several days resulted in insufficient systemic circulation volume, so her renal function deteriorated sharply. Her glomerular filtration rate was less than 5 mL/minute/1.73 m^2^. The patient did not monitor her blood sugar and was still taking metformin at the usual dose (1000 mg, orally three times a day). Based on the above factors, the lactic acidosis in this patient was thought to be MALA caused by the deterioration of renal function and metformin accumulation.

The patient had a serum creatinine of 11.22 mg/dL (which corresponds to an approximate eGFR of 3.72 mL/minute/1.73 m^2^) at the time of this admission. Two years before, her serum creatinine concentration was only 1.76 mg/dL (which corresponds to an approximate eGFR of 31.88 mL/minute/1.73 m^2^) when she was admitted to the cardiovascular department. She developed acute kidney injury due to gastrointestinal losses. The lactic acidosis was amplified by metformin use, which made her critically ill. Metformin has no antidote. Severe metabolic acidosis may be treated with hemodialysis irrespective of the underlying causes. Additionally, metformin level testing is not available in all hospitals, and measuring it is often a lengthy process. Clinicians should be mindful of the possibility of MALA in patients presenting with lactic acidosis due to other causes, such as sepsis. Metformin is a commonly used drug, and the incidence of MALA may still be underdiagnosed.

According to previous research and some case reports, blindness in MALA might be caused by severe acidosis [6–9], but the definite mechanism of transient blindness caused by acidosis is not yet known. According to some animal studies, retinal horizontal cells are very sensitive to changes in pH, undergoing uncoupling in the event of acidosis. Photoreceptor transmission to visual neurons may be interrupted, resulting in blindness [10–12].

In contrast to the unclear pathogenesis of reversible blindness in MALA, the treatment of MALA mainly consists of supportive measures such as sodium bicarbonate and other crystalloid infusions to correct the metabolic acidosis and to normalize the blood pH and lactic acid. As MALA is often mistaken for simple lactic acidosis caused by severe sepsis, these patients may often receive inappropriate antibiotics [20]. However, patients with MALA, especially those with complications such as blindness, may need renal replacement therapy (RRT). The pre-dialysis level of serum lactate is an important marker of mortality in MALA and is meaningfully higher in nonsurvivors (median 22.5 mmol/L) than in survivors (median 17 mmol/L, *p* value < 0.01) [20]. Patients with MALA should undergo dialysis without interruption until their lactate level is less than 3 mmol/L [20]. The vision loss seems to improve as soon as the metabolic acidosis is corrected [21]. In this case, after 6 hours of CVVHD, although her blood pH had not risen to normal (from 6.8 to 7.02), her vision had recovered significantly.

Unlike in other reports, this patient had stable vital signs, as her only symptoms were blindness and gastrointestinal discomfort. It may prove difficult to come to the correct diagnosis in the absence of indicative clinical features, which may delay the management of acidosis. In this case, the presence of acidosis and the patient’s history of metformin were confirmed on presentation to the emergency department, which facilitated timely management. Ophthalmic evaluation and brain imaging were also performed simultaneously to exclude other causes. To our knowledge, this is the first case report in China on transient blindness in a patient with MALA.

## Conclusions

Metabolic disease can bring about acute blindness. In patients with sudden blindness, with the presence of conditions predisposing them to acidosis, it would be prudent to manage the acidosis urgently, without waiting for the results of other examinations. Moreover, metabolic acidosis in a patient with a history of metformin intake should suggest the possibility of MALA, necessitating immediate management. Further study of reversible blindness-associated severe metabolic acidosis is needed.

MALA can occur as a complication of metformin intake, which is a common treatment of type 2 diabetes mellitus. MALA has been reported in several cases, with high mortality, and can cause reversible acute blindness. However, very few patients are referred for blindness as the chief complaint. There is also a risk of delayed treatment while ophthalmic examinations are being done in patients with relatively stable vital signs, despite presenting severe metabolic acidosis. Emergency physicians should be aware that MALA may cause acute blindness, that vision can be recovered by treatment of acidosis, and that vital signs may be stable in severe metabolic acidosis.

## Data Availability

The datasets generated and analyzed during the current study are not publicly available due to ethical concerns but are available from the corresponding author on reasonable request.
